# Commentary: Clinical characteristics and influencing factors of cardiovascular comorbidities in psoriasis—a critical appraisal of causality and shared pathogenesis

**DOI:** 10.3389/fcvm.2025.1723590

**Published:** 2025-12-01

**Authors:** Qiannan Xu

**Affiliations:** 1 Department of Dermatology, Shanghai Jiao Tong University Medical School Affiliated Ruijin Hospital, Shanghai, China; 2 Department of Dermatology, Tongji University Affiliated Shanghai East Hospital, Shanghai, China

**Keywords:** psoriasis, cardiovascular disease, influence factors, comorbidity, clinical

**A Commentary on**
Clinical characteristics and influencing factors of cardiovascular comorbidities in psoriasis By Dai X, Qin X and Li Z. (2025). Front. Cardiovasc. Med. 12:1586731. doi: 10.3389/fcvm.2025.1586731

## Introduction

We read with interest the recent article by Dai et al. (1) exploring the clinical characteristics and predictors of cardiovascular disease (CVD) in patients with psoriasis. The authors provide valuable insights into the associations between psoriasis severity (e.g., higher PASI scores), traditional risk factors (e.g., hypertension, diabetes), and CVD in a retrospective cohort of 320 patients. Their findings align with existing literature highlighting the systemic inflammatory nature of psoriasis and its potential links to cardiovascular outcomes. However, while we commend the use of robust statistical methods such as LASSO regression and multivariable logistic models, several methodological and interpretive limitations warrant discussion, as they may overstate the causal implications of psoriasis as an independent driver of CVD.

## Methodological concerns

First, the retrospective design inherently limits causal inference, a point the authors acknowledge but do not sufficiently mitigate. With data drawn from a tertiary dermatology center, selection bias is likely, as the cohort may over-represent severe psoriasis cases and those with pre-existing comorbidities (referral bias). This could inflate the observed prevalence of CVD (27.5%) and associations with factors like longer disease duration and higher PASI scores. Moreover, the broad definition of CVD—encompassing heterogeneous conditions from coronary artery disease to peripheral arterial disease, while excluding isolated hypertension—introduces outcome variability that subgroup analyses (limited by sample size) fail to address adequately. The Kaplan–Meier and Cox models, while informative, rely on retrospectively ascertained event times, potentially introducing recall or documentation bias, and the survival curves’ time scale (in months) may not accurately reflect long-term trajectories in a cross-sectional-dominant study.

A more concerning issue is the apparent inconsistencies in the reported models. In Tables 2 and 4, predictors such as “tumor stage T4 vs. T1,” “node stage N2/N3 vs. N0,” and “metastasis M1” appear alongside psoriasis-related variables like PASI and CRP. These oncology-specific terms are entirely incongruent with the study's focus on psoriasis and CVD, suggesting possible data contamination, copy-paste errors from unrelated manuscripts (e.g., cancer studies), or inconsistencies in model construction. Such anomalies undermine the validity of the multivariable results, including odds ratios (e.g., OR 2.12 for T4 vs. T1) and hazard ratios (e.g., HR 2.35 for T4 vs. T1), and raise questions about the integrity of the analysis. If these are typographical errors, they should be clarified; otherwise, they represent a fundamental flaw that calls for re-evaluation of the dataset.

Additionally, while the authors adjust for confounders like age, BMI, hypertension, and diabetes, residual confounding remains plausible. Key variables such as dietary habits, physical activity, socioeconomic status, and genetic factors (e.g., shared polymorphisms in IL-23/Th17 pathways) which might be eligible in new special study are absent, as noted in the limitations. The LASSO approach effectively handles multicollinearity but may have prematurely shrunk potentially relevant predictors (e.g., smoking, sex) to zero, overlooking their nuanced interactions. Furthermore, the observational nature precludes firm conclusions on treatment effects; the lack of a clear protective association with biologics (e.g., TNF-α or IL-17 inhibitors) is attributed to baseline severity, but without prospective randomization, this remains speculative.

## Interpretive limitations and alternative perspective

These limitations lead us to an alternative interpretation of the psoriasis-CVD relationship. We agree that psoriasis acts as an independent risk factor for CVD, as evidenced by meta-analyses showing elevated risks even after adjustment (e.g., HR ∼1.5 for severe psoriasis). However, the strength of this association appears heavily modulated by confounding factors like hypertension and diabetes, which clustered prominently in the CVD group (68.2% and 25.0%, respectively, vs. controls). Rather than portraying psoriasis as a direct causal agent in CVD progression (e.g., via the “psoriatic march” of IL-17-driven endothelial dysfunction), we posit that the two conditions often co-occur due to shared upstream causes. Both share genetic underpinnings (e.g., variants in HLA-Cw6, CARD14, and cytokine genes) and inflammatory pathways (e.g., TNF-α, IL-6, IL-23/Th17 axis), but environmental interventions—such as lifestyle modifications targeting obesity, smoking, and metabolic syndrome—likely play a larger role in modulating risk than psoriasis severity alone. In essence, psoriasis is not the proximate cause of CVD; instead, common etiologic factors (e.g., chronic low-grade inflammation from adiposity or gut dysbiosis) drive both, explaining their frequent comorbidity. Furthermore, psoriasis arthritis might also be “a missing child” among the risk factors of CVD.

## Discussion

This hypothetical assumption encourages innovative verification methods, such as Mendelian randomization to disentangle genetic causality, multi-omics integration for shared pathway mapping, or large-scale prospective cohorts incorporating wearable devices for real-time environmental exposure tracking. Dual-target therapies, like IL-23/IL-17 biologics, show promise in reducing CVD risk alongside psoriasis control (2).

By fostering such approaches, this commentary aims to propel psoriasis-CVD research toward more precise, mechanism-driven strategies for prevention and treatment. To contextualize this, we reviewed studies from the past three years (2023–2025) attempting to establish psoriasis as an independent risk factor for CVD comorbidities. These primarily include meta-analyses, cohort studies, and reviews, often relying on observational data with adjustments for confounders. However, as observational in nature, they fall short of proving direct causation due to potential residual confounding, reverse causality, and inability to establish temporal precedence definitively. Below is a Table 1 summarizing key examples:

**Table 1:** Studies focused on psoriasis as an independent risk factor for CVD disease.

Study title/reference	Type	Key claim on independence	Limitation in proving causality	Implication for shared environmental causes
The psoriasis area and severity index is an independent risk factor for cardiovascular events: a prospective register study (3).	Prospective cohort study	Higher PASI linked to CVD events after adjustment for age, sex, and traditional risks.	Observational design; potential unmeasured confounders like lifestyle; no randomization to isolate psoriasis effect.	Highlights clustering of metabolic factors (e.g., obesity, smoking), suggesting environmental exposures as common drivers.
Exploring the Link Between Genetic Predictors of Cardiovascular Disease and Psoriasis Severity (4).	Genetic association study	Severe psoriasis as independent factor in CVD development, integrated in risk scores.	Relies on genetic correlations; cannot rule out pleiotropy or environmental modifiers; lacks intervention data.	Genetic overlaps point to shared pathways, but environmental factors (e.g., diet, pollution) may amplify both via epigenetics.
Epidemiology of Hypertension in Psoriasis: An Analysis of Trends and Meta-Analysis (5)	Meta-analysis	Psoriasis comorbid with hypertension at 31.71% prevalence, implying independent risk.	Pooled observational data; heterogeneity in studies; no causal inference tools like Mendelian randomization.	Supports environment (e.g., obesity, stress) as upstream causes exacerbating both conditions through inflammation.
Progress in Cardiovascular Comorbidities of Psoriasis (6).	Review	Summarizes psoriasis as independent CVD risk based on recent cohorts.	Narrative synthesis of non-experimental evidence; selection bias in included studies.	Reviews environmental interventions (e.g., lifestyle changes) as key, indicating shared causal roles over direct psoriasis-CVD links.
All-cause and cause-specific mortality in psoriasis patients (7).	Meta-analysis	Increased CVD mortality risk in psoriasis, adjusted for confounders.	Meta of cohorts; potential publication bias; lacks mechanistic proof of direct causation.	Mortality patterns align with environmental factors (e.g., air pollution, gut dysbiosis) driving systemic inflammation in both.
A diagnostic prediction model for cardiovascular diseases (CVDs) in psoriasis patients (8).	Original research (prediction model)	Psoriasis severity in model as independent predictor for CVD risk.	Model based on retrospective data; predictive, not causal; ignores dynamic environmental influences.	Model incorporates metabolic factors, underscoring environment (e.g., diet) as common precipitants for comorbidity.

These studies, while advancing associations, underscore the limitations of observational evidence in proving psoriasis as a direct cause of CVD. Instead, they bolster the case for environmental factors—such as obesity, smoking, poor diet, air pollution, and sedentary lifestyle—as common upstream causes, which promote chronic inflammation and metabolic dysregulation, manifesting as both psoriasis flares and CVD progression. Future research should prioritize causal inference methods (e.g., Mendelian randomization, randomized environmental interventions) to test this, potentially shifting paradigms from psoriasis-centric to holistic environmental management.
